# scGENA: A Single-Cell Gene Coexpression Network Analysis Framework for Clustering Cell Types and Revealing Biological Mechanisms

**DOI:** 10.3390/bioengineering9080353

**Published:** 2022-07-30

**Authors:** Yousif A. Algabri, Lingyu Li, Zhi-Ping Liu

**Affiliations:** Department of Biomedical Engineering, School of Control Science and Engineering, Shandong University, Jinan 250061, China; algabri@mail.sdu.edu.cn (Y.A.A.); lingyuli@mail.sdu.edu.cn (L.L.)

**Keywords:** scRNA-seq, gene coexpression network analysis, cell heterogeneity, gene modules, biological mechanisms, human diabetic pancreas

## Abstract

Single-cell RNA-sequencing (scRNA-seq) is a recent high-throughput technique that can measure gene expression, reveal cell heterogeneity, rare and complex cell populations, and discover cell types and their relationships. The analysis of scRNA-seq data is challenging because of transcripts sparsity, replication noise, and outlier cell populations. A gene coexpression network (GCN) analysis effectively deciphers phenotypic differences in specific states by describing gene–gene pairwise relationships. The underlying gene modules with different coexpression patterns partially bridge the gap between genotype and phenotype. This study presents a new framework called scGENA (single-cell gene coexpression network analysis) for GCN analysis based on scRNA-seq data. Although there are several methods for scRNA-seq data analysis, we aim to build an integrative pipeline for several purposes that cover primary data preprocessing, including data exploration, quality control, normalization, imputation, and dimensionality reduction of clustering as downstream of GCN analysis. To demonstrate this integrated workflow, an scRNA-seq dataset of the human diabetic pancreas with 1600 cells and 39,851 genes was implemented to perform all these processes in practice. As a result, scGENA is demonstrated to uncover interesting gene modules behind complex diseases, which reveal biological mechanisms. scGENA provides a state-of-the-art method for gene coexpression analysis for scRNA-seq data.

## 1. Introduction

The vast majority of cells in a single organism share the same genome, although gene expression differs between tissues and cell types. One of the long-standing issues in biology and medicine is relating genotypes to phenotypes. The transcriptomic analysis is an effective way to address some of these issues [1]. One conventional method of transcriptomic analysis is an mRNA abundance measurement at the tissue or cell level and averaging it over hundreds of millions of cells in bulk RNA-seq data [2]. The bulk RNA-seq techniques have been successfully used in many studies, contributing to our understanding of gene expression [3,4]. In spite of that, the downside of bulk RNA-seq is that cell-specific mRNA abundance cannot be revealed, and biologically important gene expression regulation in individual cells may go undetected [5]. Yet, our current understanding of cell types and their dynamic alterations within biological systems is severely lacking [6]. With ongoing efforts to tackle this shortcoming, single-cell RNA sequencing (scRNA-seq) was introduced in 2009 by Tang et al. [7]. Since then, single-cell technique has been underdevelopment till it became easily accessible and was named the “method of the year” in 2014 by Nature Method [8].

The growth of scRNA-seq technology has many advantages over other existing methods, such as being a powerful technique to thoroughly characterize cellular disruption within tissues since it assesses the gene expression in individual cells [9]. Furthermore, the scRNA-seq trajectory inference technique (which means a pseudotime analysis that arranges cells along a pathway based on expression pattern similarities) can provide a detailed understanding of dynamic cell differentiation [10]. Moreover, scRNA-seq datasets are primarily utilized to identify cell types or to discover new biomarkers. Gene expression in a single cell is considered stochastic, so the gene expression values and their interactions in different cells vary enormously [11,12]. However, it is significant to develop a comprehensive understanding of these interactions between components and coordination in the gene expression of an organism [13].

Gene coexpression networks (GCNs) have proven particularly useful in identifying relationships and annotating functions of uncharacterized genes [14,15,16]. GCNs are commonly developed to identify phenotype-specific biomarkers that contain genes with functional associations based on coexpression relationships [17,18,19].

Although there is a rapid increase in available tools to analyze scRNA-seq data, no systematic pipeline comprehensively analyzes scRNA-seq, including constructing a gene coexpression network analysis. There are some accessible packages for GCNs, such as weighted gene coexpression network analysis (WGCNA), coexpression of RNA-seq data (Coseq), and coexpression modules identification tool (CEMiTool), petal, CoXpress, and coexpressed biological processes (CoP) [20]. However, they were initially designed to analyze microarray and bulk RNA-seq datasets [21,22]. Furthermore, these GCNs packages cannot be directly used for analyzing single cells because of the sparse data in the scRNA-seq data. To the best of our knowledge, there is no complete systematic pipeline including all the above analysis procedures.

Therefore, this study aims to illustrate a single-cell gene coexpression network analysis (scGENA) framework in a systematic pipeline to analyze scRNA-seq data. scGENA aims to implement a complete R software package using scRNA-seq data with a step-by-step guide for the entire analysis, including data preprocessing, differential gene expression, data imputation, construction of gene coexpression networks, and investigating key gene modules that enrich critical functions in diverse cell types.

## 2. Materials and Methods

### 2.1. Overview of scGENA

**Figure 1:**
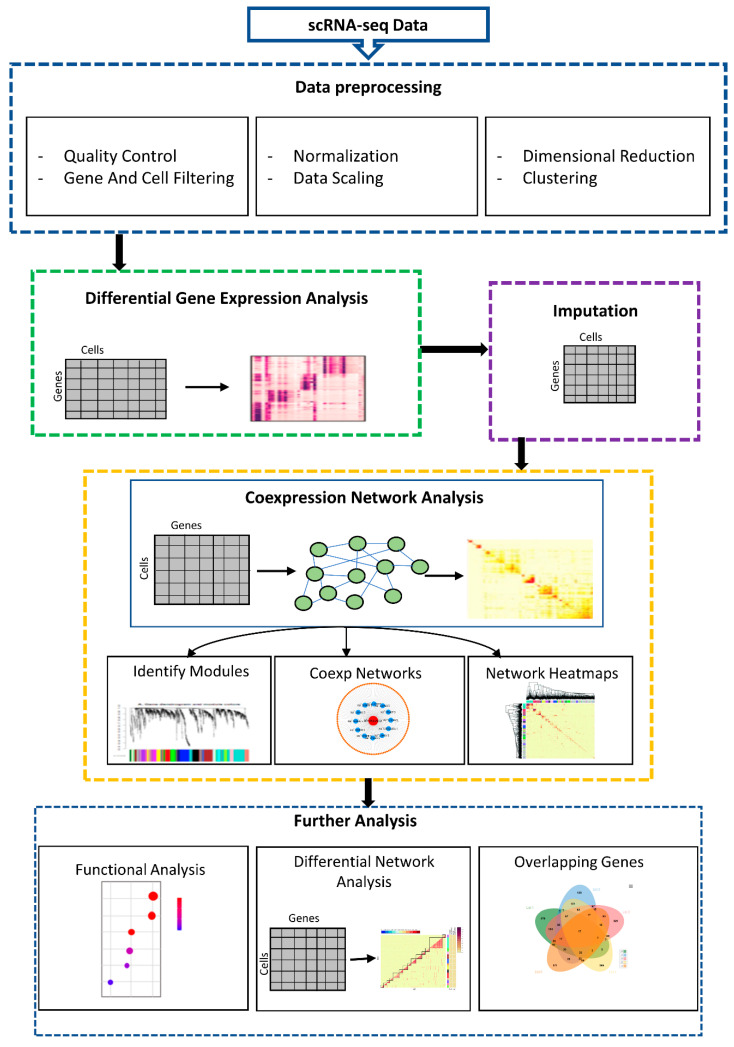
Flowchart of scGENA pipeline.

scGENA is a systematic pipeline for single-cell data analysis and contains five phases, as illustrated in Figure 1. *Phase 1* set up and preprocesses the scRNA-seq dataset to filter low-dimensional and noisy single-cell expression genes. *Phase 2* performs a differentially expressed (DE) genes analysis to determine which genes are expressed significantly different in different conditions. These genes can reveal biological information about the processes that are influenced by the conditions of interest. *Phase 3* applies the SAVER imputation method to estimate and replace dropout values in each gene cross cell’s actual missing expression level, reducing technical differences while preserving biological variability across cells [23]. *Phase 4* constructs a coexpression network analysis to shed light on the transcriptional regulatory mechanisms underpinning numerous biological processes [24]. *Phase 5* performs further analyses, including a functional enrichment analysis, differential coexpression network analysis, and overlapping genes identification across different cell-types to better interpret the biological insights. scGENA is fully implemented in R and available at GitHub (https://github.com/zpliulab/scGENA) (accessed on 1 September 2020). The following subheadings describe the details of each phase of scGENA.

### 2.2. Preprocessing of scRNA-seq

In the first phase, scGENA sets up and preprocesses the scRNA-seq dataset. The single-cell data input is a matrix composed of genes as rows and cells as columns, containing the counts of gene expression in every cell.

This study implemented a human pancreas with a nondiabetic and type 2 diabetic disease dataset as case studies for illustration purposes. The dataset contained 1600 cells aggregated from 12 nondiabetic and 6 T2D organ donors of cell types of α-, β-, δ-, and PP-cells using the Fluidigm C1 cell-capturing process and 39,851 features (the human whole-genome sequencing including genes and ncRNA transcripts), as summarized in Table 1. We downloaded the data from the NCBI Gene Expression Omnibus (GEO) repository with accession ID GSE81608. After the data preparation, we used the Seurat method (https://satijalab.org/seurat/index.html (accessed on 1 September 2020)) for quality control, normalization, data exploration, and visualization of the preprocessing steps [25].

### 2.3. Differential Expression (DE) Analysis

Differential expression (DE) analysis is one of the most common tasks for scRNA-seq data. Although there are well-established techniques for such research in bulk RNA-seq data, tools for scRNA-seq data are still in the early stages [27]. We employed the R/Bioconductor MAST (model-based analysis of single-cell transcriptomics) package for this analysis based on empirical tests, which build two-part generalized linear models designed explicitly for bimodal and zero-inflated single-cell gene expression data [28,29]. MAST accounts for dropout events using a hurdle model while modeling variations in gene expression based on condition and technical variables. The hurdle model improves differential gene expression by summarizing differences between two groups with pairs of regression coefficients [28]. In this process, the highly differentiated genes were evaluated across the resulting four cell types and divided into clusters; the adaptive threshold was a cut-off value selected based on the gene’s median expression value (in this case, we limited ourselves to genes that expressed in at least 0.25 within the cells). It also determined a single cluster’s positive and negative expressed markers. We then selected the DE genes in each cell type to construct the gene coexpression networks and for further downstream analysis. In total, 2169 genes were selected as differentially expressed and presented in the Supplementary Materials Table S1.

### 2.4. Data Imputation

Due to the low transcript abundances in single cells, current scRNA sequencing technologies may fail to detect some gene expression. This can result in missing expressed gene values, known as a dropout event [30]. Dropouts can potentially cause significant bias in gene–gene correlations and other downstream analyses [31]. Recently, imputation methods have been developed to estimate actual expression levels directly. Here, we used the SAVER method in this pipeline because it imputed original zero values to actual values [32]. SAVER estimates expression levels by borrowing information across genes and applying a Bayesian technique. The main reason to use SAVER imputations is that it uses gene-to-gene relationships to impute the values of each gene expression level in each cell. It reduces technical variation while maintaining biological variability between cells [23].

### 2.5. Gene Coexpression Networks (GCNs) Analysis

The novel ensemble scGENA framework for constructing single-cell-based GCNs is based on combining multiple methods to establish a systematic R-based pipeline. Previous tools and packages for building GCNs were designed to analyze bulk RNA-seq and microarray data. Therefore, because single-cell data are intrinsically noisy and sparse, traditional measures (such as Pearson, Spearman, or cosine correlation) cannot be used effectively [33]. In contrast, mutual information (MI) has significant advantages over other measures because it may capture complex nonlinear and nonmonotonic interactions and represent the dynamics of groups or pairs of genes [34,35]. MI approaches are, therefore, often the preferred method for such a network inference analysis. We employed the *minet* package to discretize and compute the distance for the mutual information matrix as follows:

(1) $$MIM_{ij}=I\left(X_{i\;};X_{j}\right)$$

where i, j is the MI between $X_{i\;}$ and $X_{j}$, and $X_{i\;}\epsilon \;\chi$, i=1,…,n, is a discrete variable representing the ith gene’s expression level. Additionally, the empirical estimator is selected to estimate the amount of information shared by any pair of genes due to its ability to decrease the bias without affecting variance [36].

## 3. Results and Discussion

In this paper, we used the transcriptomic data of human pancreas cells from [26] to build an integrated systematic pipeline for a complete analysis of scRNA-seq data, including data exploration, quality control, normalization, dimensional reduction, a cell clustering, differential genes analysis, a gene coexpression network analysis, and a further downstream analysis.

### 3.1. Data Preprocessing

**Figure 2:**
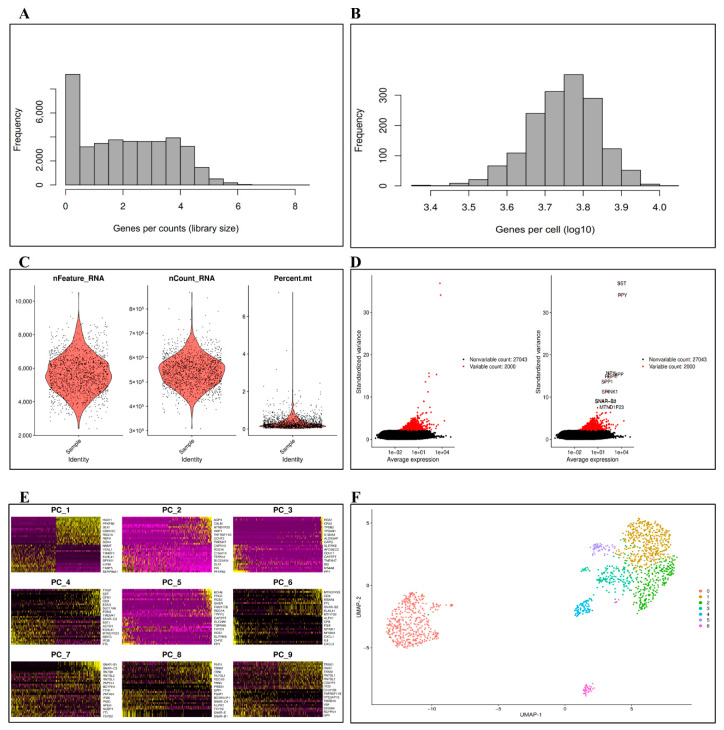
Preprocessing of scRNA-seq data. (**A**) Measurement of library size for genes per counts; (**B**) Genes distributions among cells; (**C**) Quality control; (**D**) Variable genes in the dataset; (**E**) Dimensionality reduction heatmap PCA; (**F**) UMAP clustering of the dataset.

We first explored the data library size and the distributions of genes within different cells for 1600 samples of human donors α-, β-, δ- and PP cells from nondiabetic and T2D organ in the preprocessing phase, as shown in Figure 2A,B. This step is necessary because it enables researchers to understand the dataset comprehensively. Next, we employed Seurat for data quality control (QC) and visualization. Seurat aims to detect and evaluate heterogeneous sources of single-cell transcriptomic measurements and dataset integrations [25]. The QC was performed to exclude cells with <1500 or >10,000 expressed genes and with >15% of unique molecular identifiers (UMIs), as well as the contaminated cells. Therefore, the number of remaining single cells was 1472, with 29,043 and variable genes of 2000 selected for the downstream analysis by calculating a group of genes in the dataset with a significant level of cell-to-cell variation (using FindVariableFeatures, as a Seurat function).

The quality control, variables, and nonvariable genes of single-cell data are displayed in Figure 2C,D. The data are then logarithm-normalized by employing the LogNormalize technique, which is regarded as a global normalization method that divides the gene counts for a single cell and then multiplies by the scale factor. The result was then transformed with the natural log using log(x + 1) to account for zero counts. The normalization process is essential for uncovering a dataset’s underlying biological heterogeneity. The normalization approaches are also important to prevent noise and bias and are necessary for dimensionality reduction [37].

These upstream analysis procedures, such as quality control filtering and normalization, can significantly affect clustering and trajectory inference. Following the completion of the preprocessing procedures, the subsequent analytical phases, which included dimensionality reduction, clustering, and trajectory inference, focused on discovering patterns in the data that gave biological insights. Dimensionality reduction reduces the dataset to a more compact and potentially interpretable representation that enables researchers to capture the key biological axes of variation and enhances clustering and trajectory inference performance [38]. Figure 2E illustrates the dimensionality reduction plotted by a principal component analysis (PCA) heatmap and gene clustering across cells by UMAP (uniform manifold approximation and projection). The PCA, which provides a linear combination of genes that best reflects the variation in the data, is the most often used dimensionality reduction approach for scRNA-seq analysis. The PCA’s ability to lessen data dimensionality while identifying the dimensions with the most variance makes it a useful dimensionality reduction method prior to clustering [39]. Figure 2F visualizes the gene clusters in the dataset.

### 3.2. DEs and Imputation

**Figure 3:**
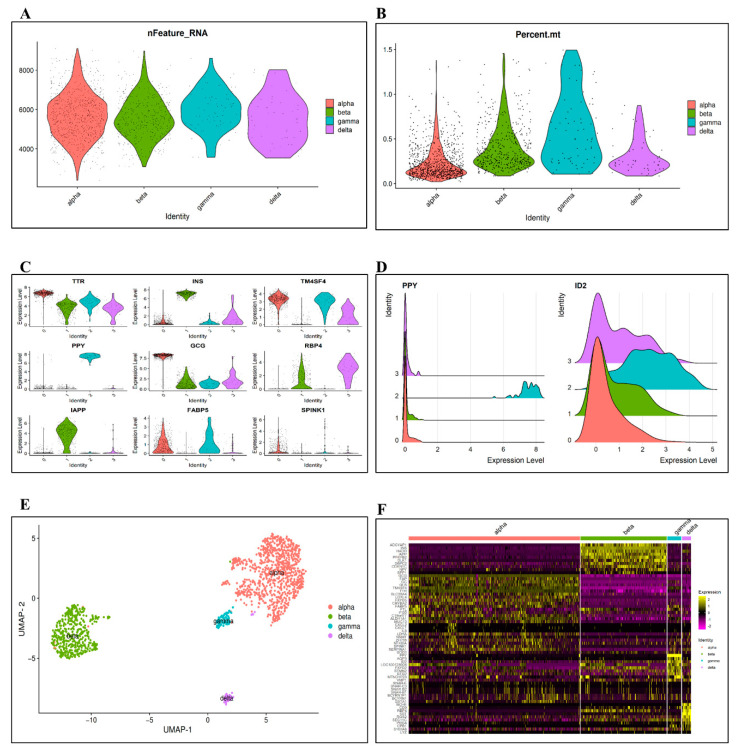
Differential expression analysis in scGENA. (**A**) Gene clustering and distribution in all data cell-types. (**B**) Gene distribution based on the percentage of mitochondria. (**C**,**D**) Highly differentiated genes. (**E**) Cell-types clustering using UMAP. (**F**) Heatmap showing the level of gene expression in all cell-types.

The field of biomedical research has entered the omics era with the emergence of high-throughput technology, and research tools focused on bulk RNA-seq data no longer meet this development’s objectives. However, dealing with such a massive volume of data presents significant difficulties in extracting and analyzing data. Sequencing data analysis frequently yields a list of genes that are differentially expressed. However, it is challenging for many researchers to connect the vast number of differential genes or proteins to a biological event to be examined. We need to group genes with similar expressions and associate them with their biological phenotypes using DE tools to study the functional enrichment analysis for these genes. In our pipeline, we used Seurat’s MAST method to perform a differential gene expression analysis based on a generalized linear model [28]. We selected the differentially expressed genes based on the criteria previously mentioned (see Section 2.3), which necessitate a gene being identified at the cut-off threshold in either of the two cell groups and being differentially expressed (on average) by some proportion between the two different cell groups, see Supplementary Materials Tables S1. In a study [40], MAST performed as the best single-cell DE testing technique, outperforming bulk and single-cell approaches in a small-scale comparison on a benchmark dataset [41,42]. Figure 3A presents the DE genes based on the number of genes detected in each cell, while Figure 3B shows how to classify cells depending on the presence of mitochondrial genes. Out of these DE genes, we selected the top 10 from each group for further analysis based on the logarithm fold-change threshold of the average gene expressions. These genes are visualized in Figure 3C,D. We then clustered all the DE genes for each cell type using UMAP and heatmap plots, as depicted in Figure 3E,F.

**Figure 4:**
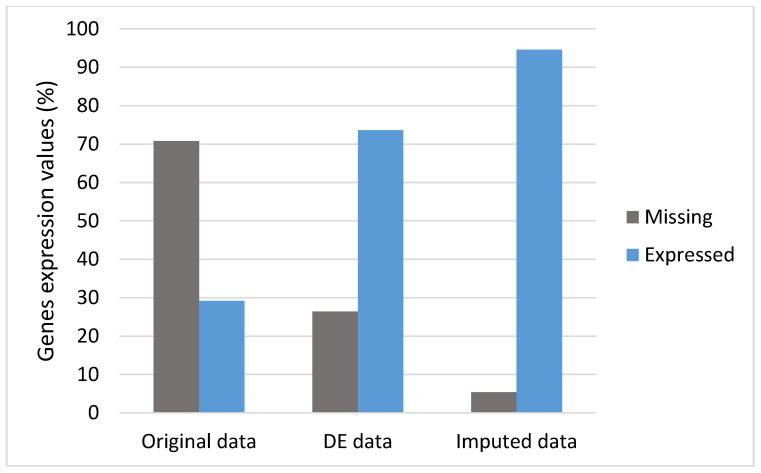
Comparing the data before and after performing imputation.

An imputation method was performed to impute the missing gene expression values in the count matrix to reduce the effect of noise and dropout events. In this pipeline, we used the SAVER method, which showed good performance for imputing most of the missing values. Figure 4 compares the difference before and after imputation for the selected 2000 genes from the DE step across 50 samples. It dramatically imputed gene expression compared to original and differentially expressed genes. As can be noticed in Figure 4, the level of nonzero expression values changed from 28% to 94.6% after imputation. Therefore, we used these imputed data for our downstream analysis.

### 3.3. Gene Coexpression Networks Analysis

GCN is an approach for inferring gene module function and gene-disease interactions from genome-wide gene expression. This method builds networks of genes with a tendency to coactivate across a group of samples and then interrogates and analyzes this network [43]. GCNs can be employed for various purposes, including candidate disease gene selection, functional gene identification, and gene regulation discovery.

**Figure 5:**
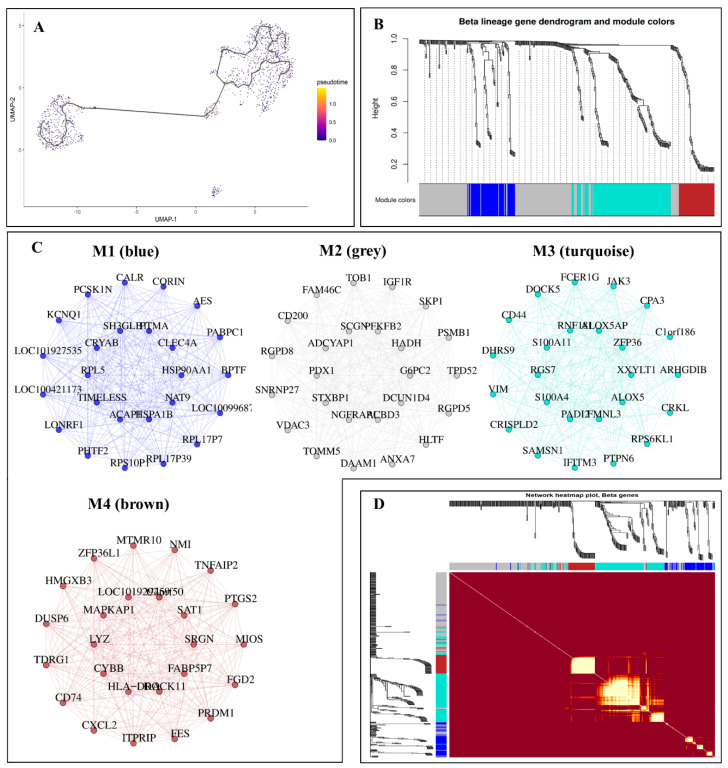
Coexpression analysis in scGENA. (**A**) Pseudotime trajectory of the cell types; (**B**) Dendrogram and modules colors clustering for β-cells; (**C**) Gene coexpression networks for the four modules in β-cells (M1, M2, M3, and M4); (**D**) Coexpression network heatmap.

Since WGCNA was initially designed to analyze bulk RNA data [44], its performance on single-cell data is limited because of the inherent sparsity of scRNA-seq data [21,22]. To resolve this, our pipeline has a function that aggregates the transcriptionally similar cells into a pseudobulk cell type before running WGCNA in our framework. Figure 5A shows the pseudotime aggregation for the four cell types of this dataset. However, because of the MI symmetry characteristic, it relies on the pseudotime input to infer the GCNs [45]. Then, we utilized a signed consensus network based on the WGCNA algorithm for a particular cell type (see [46,47]), computing component-wise values for topological overlap in the dataset. Biweighted midcorrelations (defined in WGCNA as *bicor*) were computed for each pair of genes, followed by a signed similarity matrix. The similarity between genes in the signed network showed the sign of the connection of their expression patterns.

On an exponential scale, the signed similarity matrix was then boosted to power β, varying the cell types to accentuate strong correlations and lessen the emphasis on weak correlations. The selection of the power β or soft thresholding, as called in the WGCNA package, was based on the number of data samples (see [46]). The resulted adjacency matrix was then turned into a topological overlap matrix. Figure 5B,C visualize the hierarchal clustering tree (dendrogram) and gene coexpression networks of β-cells data. We have selected only 25 genes for each module to visualize a clear network. Therefore, modules were formed by utilizing module-cutting criteria such as a minimum module size of 100 genes, with a deepSplit score of 4, and a correlation threshold (mergCutHeight) of 0.2 that can be used to merge modules. Modules having a correlation larger than 0.8 were combined. These parameters were selected to construct the block-wise networks based on the samples’ data sets. As a result, four coexpression modules were significantly correlated in β-cells; therefore, these modules’ genes were used for the functional enrichment analysis. Figure 5D depicts the gene networks for β-cells module genes using a heatmap. The heatmap of each row and column correspond to a single gene and the relationship between genes. Module colors and gene dendrograms are also plotted on the left and top sides of the heatmap. For the construction of gene coexpression networks for the other three cell types and their identified modules, see Supplementary Material Figures S1–S3.

### 3.4. Further Analysis

**Figure 6:**
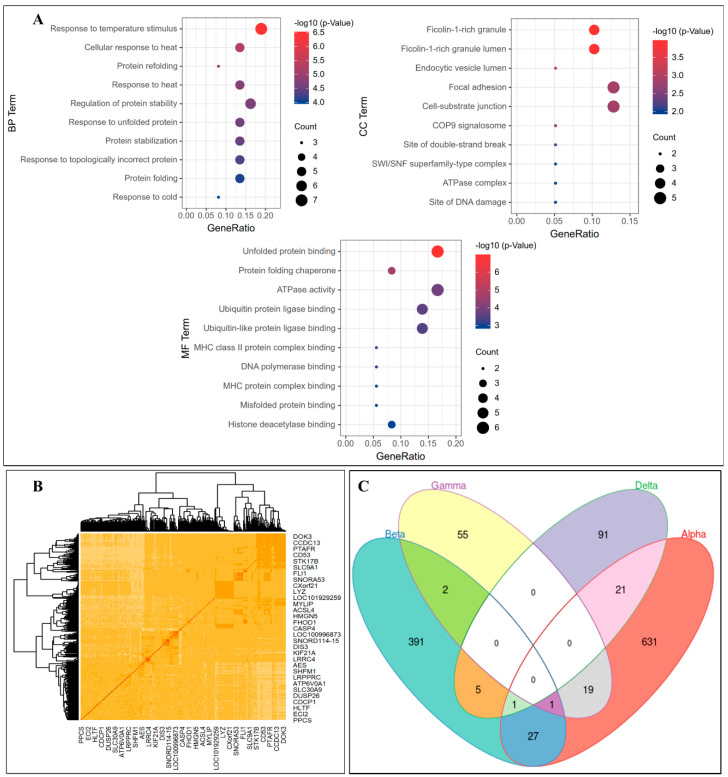
Further analyses in scGENA. (**A**) The significantly enriched GO terms for the β-cells for BP, CC, and MF. (**B**) Differential coexpression heatmap by MODA. (**C**) Genes overlapping among the four cell types by the Venn diagram.

In this substep, we performed a functional enrichment analysis, differential coexpression analysis, and the identification of overlapping genes across the cell types for co-expression network genes in each module to investigate the biological insights. A functional enrichment analysis is specifically used to identify the gene sets linked with a biological process or molecular function in order to interpret the underlying physiological insights and reveal the dysfunctional mechanisms. Therefore, we carried out a Gene Ontology (GO) terms enrichment using the R package ClusterProfiler, which included the following three categories: biological process (BP), cellular component (CC) and molecular function (MF). In this analysis, we demonstrated the top ten terms within each category that were considerably enriched significantly in M1, as illustrated in Figure 6A. A subset of each enriched term was chosen and shown as a network plot, with terms with similarity > 0.3 linked by edges with the best p-values from each cluster to further understand the relations among the terms. All network modules’ GO terms listed are presented in Supplementary Materials Tables S2–S12.

The essential GO terms in the three categories were, respectively, protein folding (GO:0006457) for BP, the cell-substrate junction (GO:0030055) for CC, and unfolded protein binding (GO:0051082) and protein folding chaperone (GO:0044183) for MF. The study of [48] emphasizes that high inflammatory cytokines might induce the accumulation of unfolded or misfolded proteins, i.e., endoplasmic reticulum stress in diabetic pancreatic islets. In the CC category functional enrichment investigation, Zhang et al., 2022 [49] discovered that the upregulated differentially expressed genes concerning acute pancreatitis were connected with the cell–substrate junction. On the other hand, chaperones’ adaptive unfolded protein response signaling maintained endoplasmic reticulum protein folding equilibrium in healthy beta cells, according to Yong et al., 2021 [50]. Figure 6A shows that several of our targeted genes included protein folding terms. We assumed that the BP and MF protein-folding-related GO terms were relevant in pancreatic or diabetes disease. Furthermore, several of our identified genes were enriched for the BP and MF categories, with only a few enriched for CC. The enhanced GO terms may be crucial in revealing the progression of diabetes disease.

Once the coexpression gene modules are defined, researchers can utilize these findings to perform several analyses, such as a differential coexpression network analysis. Consequently, we used MODA for the differential coexpression network analysis [51], which can analyze the different networks of each cell type of data. In order to reduce the number of modules as much as possible, the gene was sampled; in other words, only differential genes were selected as the input, as shown for the beta cells in Figure 6B. The other cell types’ results are shown in the Supplementary Materials Figures S4–S6. Last but not least, we identified the consistent and overlapped genes across all four cell types using the *intervene* R package [52] to plot a Venn diagram, as shown in Figure 6C. It is observed that beta–alpha cells share many genes compared to other cell types. Identifying these overlapping genes will assist the researcher in learning more about human islet cell biology and pathophysiology, particularly the alpha-cell-derived paracrine signals’ role in normal beta-cell survival and function [53]. The experimental analysis in the study [53] on human and mouse islet cells indicated that the crucial variable was not necessarily a different species but the differing alpha–beta-cell ratio. The set point of glucose homeostasis in the body appears to be determined by paracrine interactions between alpha and beta cells. It is still unclear how the various islet designs found in different animals connect to their glycemic set points.

Furthermore, suppose specific genes in scRNA-seq data consistently exhibit identical expression changes in biological processes or various tissues. In that case, we suspect these genes are functionally associated and may be categorized as a module. We can utilize the findings of the gene modules to perform many different analysis tasks.

## 4. Conclusions

Advances in scRNA-seq technology have resulted in the generation of datasets with increasing size and complexity. As a result, an ecosystem of computational approaches has been developed to address the issues associated with evaluating big datasets. In this work, we proposed an integrative pipeline scGENA for a complete single-cell gene coexpression analysis based on scRNA-seq data. scGENA integrates numerous models to comprehensively perform several steps: preprocessing, dimensionality reduction, clustering, differential genes identification, imputation, and network construction analysis. Because scRNA-seq data are often sparse and noisy, it is challenging to build coexpression and differential coexpression networks. We showed how to use the scGENA framework to construct and analyze coexpression networks using scRNA-seq data in an integrative and reliable way. The results demonstrated that the scRNA-seq-based method was good and valuable for identifying cell types and revealing biological insights by analyzing gene coexpression patterns.

## Figures and Tables

**Table 1:** Summary of single-cell data information in the proof-of-concept study.

GEO No.	Type of Cells	Cells	Features	Organism	Protocol	Ref.
GSE81608	α- β- δ- PP	886 472 49 85	39,851	Homo sapiens	SMARTer	Xin et al., 2016 [26]

## Data Availability

The single-cell data used in the study can be obtained from Gene Expression Omnibus with accession number: GSE81608.

## Supplementary Materials

The following supporting information can be downloaded at: https://www.mdpi.com/article/10.3390/bioengineering9080353/s1, Figure S1: Gene co-expression networks in Alpha; Figure S2: Gene co-expression networks in Delta; Figure S3: Gene co-expression networks in Delta; Figure S4: Differential co-expression heatmap for correlated genes in Alpha cells; Figure S5: Differential co-expression heatmap for correlated genes in Delta cells; Figure S6: Differential co-expression heatmap for correlated genes in Gamma Cells; Table S1: Differential genes expressions; Tables S2–S12: Gene Ontology for the four cell types.
