# Supplementary material for: scGENA: A Single-Cell Gene Coexpression Network Analysis Framework for Clustering Cell Types and Revealing Biological Mechanisms
Source: Bioengineering (Basel). 2022 Jul 30;9(8):353. doi: 10.3390/bioengineering9080353 (PMC9405199; doi:10.3390/bioengineering9080353)
Supplement: Supplementary file 1 [file bioengineering-09-00353-s001.zip › bioengineering-1788406-supplementary.pdf]

Supplementary Materials

scGENA: A single-cell gene co-expression network analysis framework for clustering cell types and revealing biological mechanisms

Yousif A. Algabri [1], Lingyu Li [1] and Zhi-Ping Liu [1]*

[1]Department of Biomedical Engineering, School of Control Science and Engineering, Shandong University, Jinan, Shandong 250061, China; algabri@mail.sdu.edu.cn, lingyuli@mail.sdu.edu.cn, zpliu@sdu.edu.cn

*Correspondence: zpliu@sdu.edu.cn

1. Co-expression Networks construction

These networks are constructed for only randomly selected 25 genes in each cell types modules.

Alpha-cells:

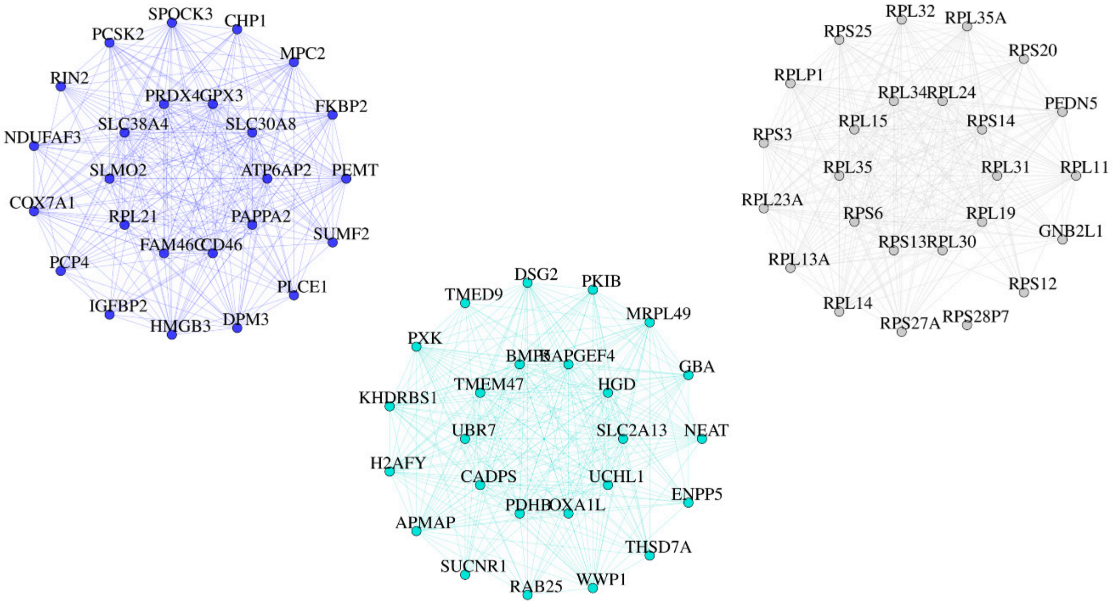

Figure S1: Gene co-expression networks in Alpha.

Delta-Cells

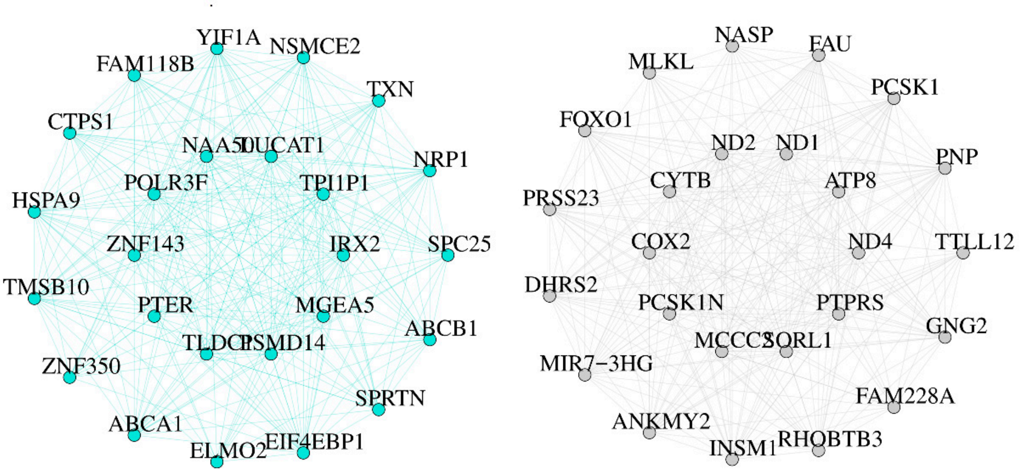

Figure S2: Gene co-expression networks in Delta.

Gamma/PP Cells

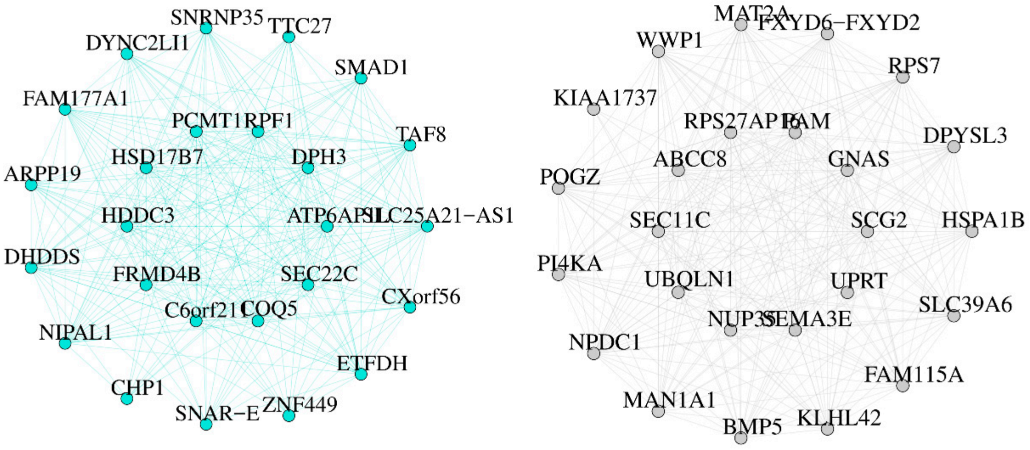

Figure S3: Gene co-expression networks in Delta.

2. Differential Co-Expression Analysis

In this step, we identify the differential co-expression genes in all cells type in the islet human pancreatic data. The heatmaps are plotted by MODA package in R.

Alpha genes heatmap:

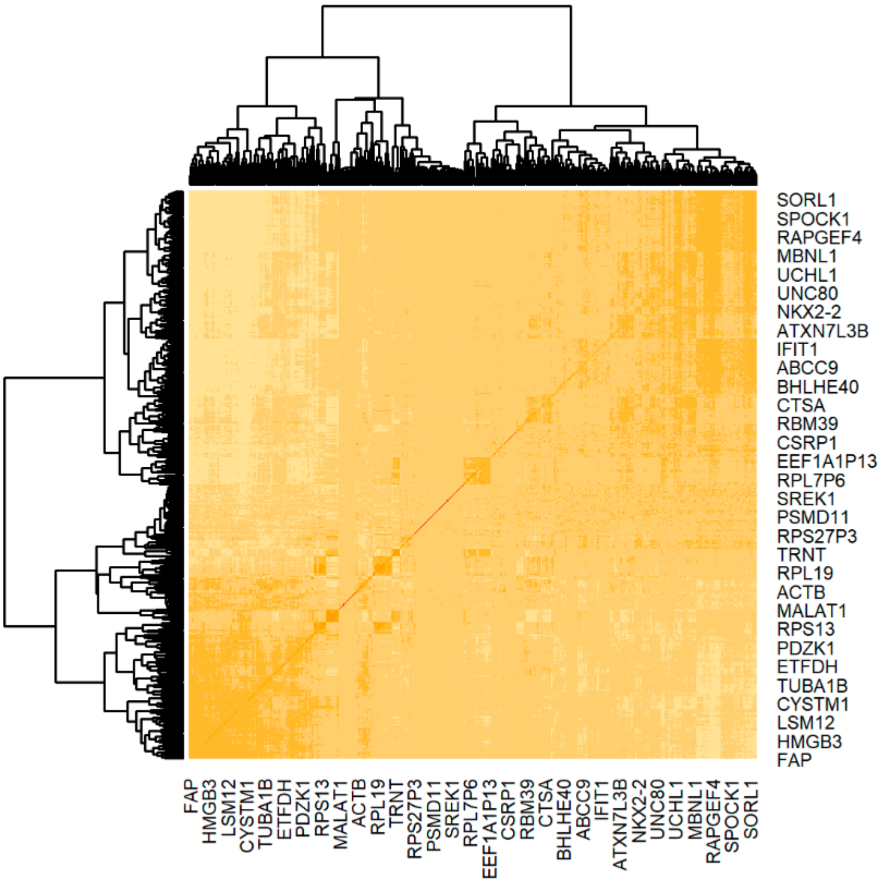

Figure S4: Differential co-expression heatmap for correlated genes in Alpha cells.

Delta genes heatmap:

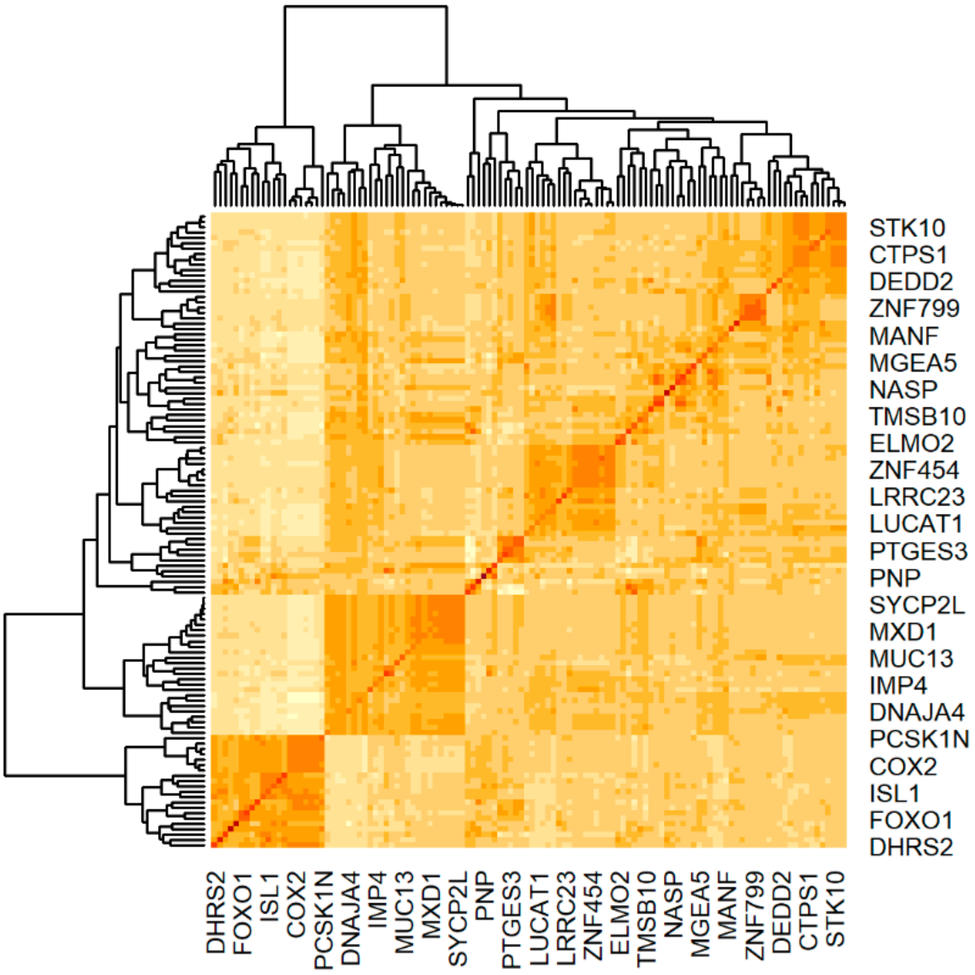

Figure S5: Differential co-expression heatmap for correlated genes in Delta cells.

Gamma genes heatmap:

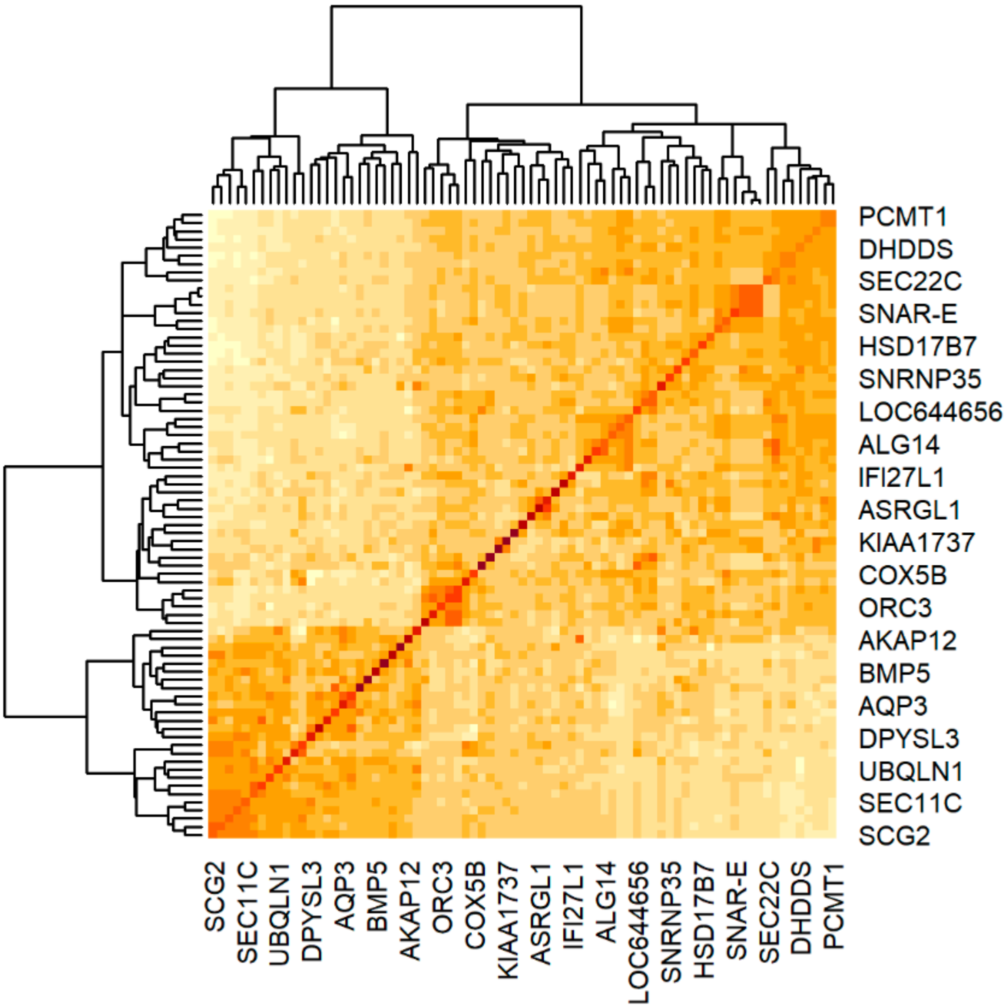

Figure S6: Differential co-expression heatmap for correlated genes in Gamma Cells.
